# Alcohol consumption and internalising disorders in young adults of ALSPAC: a population-based study

**DOI:** 10.1136/jech-2020-213922

**Published:** 2020-07-06

**Authors:** Gwen Sascha Fernandes, Gemma Lewis, Gemma Hammerton, Kushala Abeysekera, Liam Mahedy, Alexis Edwards, Glyn Lewis, Matthew Hickman, Jonathan Heron

**Affiliations:** 1 Population Health Sciences, University of Bristol School of Social and Community Medicine, Bristol, UK; 2 UCL Division of Psychiatry, London, UK; 3 Virginia Institute for Psychiatric and Behavior Genetics, Richmond, Virginia, USA; 4 School of Social and Community Medicine, University of Bristol, Bristol, UK

## Abstract

**Introduction:**

Depression and harmful alcohol consumption contribute significantly to the global health burden, but in young adults, this relationship is under-researched and conflicted. The aim of this study was to determine the sex-based prevalence and the association between internalising disorders such as depression and alcohol use disorders.

**Method:**

Using the Avon Longitudinal Study of Parents and Children, we assessed the sex-specific prevalence of International Classification of Diseases, Tenth Revision diagnosed generalised anxiety disorder (GAD), depression and fear-based anxieties (FBA) at 24 years (n=3572). We examined the association between internalising disorders and alcohol consumption using the Alcohol Use Disorder Identification Test for Consumption 5+ threshold and Diagnostic and Statistical Manual on Mental Disorders defined criteria for alcohol dependence.

**Results:**

Women reported more GAD (11.6% vs 6.5%), depression (13.4% vs 6.9%) and FBA (1.3% vs 0.5%) than men (p<0.001). Harmful drinking, after adjustment for sex and socioeconomic status, was associated witha higher prevalence of depression (OR 1.8, 95% CI 1.3 to 2.4, p<0.001), anxiety (OR 1.4, 95% CI 1.0 to 2.0, p<0.001) and FBA (OR 2.4, 95% CI 1.04 to 5.56, p=0.009) compared with lower-risk drinkers. In contrast, hazardous drinking was associated with a lower prevalence of GAD (OR 0.69, 95% CI 0.54 to 0.88) and depression (OR 0.68, 95% CI 0.54 to 0.86) compared with lower-risk drinkers.

**Conclusions:**

Young adults in the UK who drink harmfully are more likely to have depression and other internalising disorders. Further research should test whether there is a J-shaped relationship between alcohol consumption and mental health in young people and whether this varies across the life course.

## INTRODUCTION

Early adulthood (aged 18 years and over) is a developmental period marked by seminal life changes (eg, leaving home, finding a job, etc) and is considered a high-risk period for the onset of internalising disorders and/or alcohol-related problems.^1–3^ In the UK, excessive alcohol use remains the leading cause of premature mortality and can have negative consequences on mental health particularly an increased risk of depression and anxiety.^4^ Previous studies have shown consistent associations between alcohol use disorders (AUD) and major depressive disorders.^5–7^ This is due in part to alcohol consumption leading to depression, and individuals with depressive symptoms being more likely to consume alcohol frequently and in higher quantities—the ‘self-medication’ hypothesis.^8^ Some older adult, non-UK-based studies indicate that alcohol misuse and anxiety/depressive symptoms are extensively associated with each other, although the nature of the association is often inconsistent by gender or age groups.^3 9 10^ In the UK, results from the Whitehall II prospective cohort study found that mental health is the primary indicator of change in the relationship between mental health and alcohol consumption in middle-aged adults. Poor mental health predicted increasing alcohol consumption among lower-risk drinkers and was a maintaining factor for heavy alcohol consumption later along the life course.^11^ The conflicting evidence has been attributed to small sample sizes; heterogenous sample characteristics (university vs general populations); variations in study designs; or varying assessments of alcohol use and severity of use (validated tools and thresholds).^2 3 10^

There is a need for evidence on the relationship between internalising disorders and alcohol consumption from a large contemporary general population data set, specifically among young adults. Given this under-researched and controversial topic,^12 13^ our aim was to investigate the burden of drinking and internalising disorders in a young adult, community-based sample in the UK. The study will use data from the Avon Longitudinal Study of Parents and Children (ALSPAC) and first, aims to presents sex-based prevalences of internalising disorders such as anxiety and depression and alcohol use in 24-year-olds. Second, the study will test the association between these internalising disorders and alcohol consumption with adjustment for potential covariates such as sex and socioeconomic factors.

## METHODS

### Sample

Data were drawn from ALSPAC, the most phenotyped birth cohort in the world, and detailed information about our ALSPAC sample is presented as online supplementary material 1.

Of the 10 018 ALSPAC study participants who were invited for a clinical assessment, 40.1% (n=4021/10 018) attended for clinical assessment (denoted TF5 visit) (mean age of 24 years (22–26 years; SD 0.8; 1507 males, 2510 females^14^). Informed consent for use of data collected via questionnaires and clinics was obtained from participants following the recommendations of the ALSPAC Ethics and Law Committee at the time.

A flow chart of participants in this study is presented in online supplementary appendix 1.

## MEASURES

### Internalising disorders

#### Generalised anxiety disorder (GAD)

GAD was assessed using a self-administered online version of the Clinical Interview Schedule-Revised (CIS-R) based on the extensively used International Classification of Diseases,Tenth Revision (ICD-10) criteria^,15 16^ and created a binary variable indicating presence or absence of GAD over the past 1-week period.

#### Depression

We assessed depression using the CIS-R and ICD-10 criteria based on the previous 1-month period.^15^ For this study, we used a binary variable indicating the presence of depression (merged mild, moderate and severe depression) or not over the past 1-week period.

#### Fear-Based Anxieties (FBA)

Given that social anxieties associate with harmful and hazardous alcohol consumption,^17 18^ we created a binary FBA variable by grouping three ICD-10 anxiety disorders from CIS-R: agoraphobia, social phobia and panic disorder over the past 1 week due to interrelated and overlapping cluster of phobic symptoms between them.

The use of this standardised version of the CIS-R within a community setting such as ALSPAC has been previously validated and demonstrated that lay assessors such as research nurses were as reliable as psychiatrists and did not exhibit bias in their use of CIS-R.^19^ The CIS-R assigns a total weighted score according to the frequency and severity of symptoms during the week immediately preceding the interview.

### Alcohol measures

Within the ALSPAC sample, alcohol problems were assessed from responses to a computer-assisted standardised clinical interview. We used Alcohol Use Disorder Identification Test for Consumption (AUDIT-C) to assess hazardous drinking in the past 1 year.^20 21^ With the AUDIT-C data, we used a validated (≥5) cut-point to derive a binary measure as an indication of hazardous alcohol use.^22^ ALSPAC also collected data on alcohol use and alcohol dependence on administering using the Diagnostic and Statistical Manual on Mental Disorders—Fourth Edition (DSM-IV) tool (consisting of four items of alcohol abuse and seven items of alcohol dependence) and refers to drinking behaviour in the past 1 year. This measure of AUD comprises all individuals who indicate alcohol abuse and/or alcohol dependence, a maladaptive pattern of alcohol use leading to clinically significant impairment or distress and corresponds with harmful drinking behaviour that causes serious consequences physical and mental health.^23 24^

The Office for National Statistics (2018) reports that almost half the population between the ages of 16–24 years consumed alcohol in the past week and of these, almost 30% of all drinkers exceeded the recommended alcohol units on the heaviest drinking days. Given these findings, heavy drinking to a hazardous level in a young adult population^25^ is not uncommon and needs to be separated from more harmful drinking patterns—indicating alcohol abuse or alcohol dependence. Therefore, we created a three-category alcohol phenotype (lower-risk drinkers, hazardous drinkers and harmful drinkers) by combining the AUD with (AUDIT-C). The following three drinking categories were generated:

1. AUDIT-C <5 and no AUD=lower-risk drinkers,
2. AUDIT-C ≥5+ no AUD=hazardous drinkers and
3. AUDIT-C ≥5+ AUD-positive=harmful drinkers.

A sensitivity analysis was undertaken in which an alternative classification of alcohol use was created where all individuals with an ‘extremely hazardous’ AUDIT-C drinking score (10–12) were included in the harmful drinker category. The following three categories were used:

1. AUDIT-C <5 and no AUD=lower-risk drinkers,
2. AUDIT-C ≥5–9+ no AUD=hazardous drinkers and,
3. AUDIT-C ≥10 OR AUD-positive=harmful drinkers.

### Statistical analysis

Prevalence of internalising disorders and alcohol use was presented by gender, and associations were tested using Pearson’s χ^2^ test (with associated p value). ORs and their corresponding 95% CIs were derived from logistic regressions with the lower-risk drinking group as the referent. We presented a crude model and models adjusted for sex (model 2) and for sex and socioeconomic status, parental income and maternal education (model 3) using imputed data. Further multiple imputation details are in online supplementary material 2. We used STATA 15.1 for all our analyses.

## RESULTS

A total of 3572 participants attended for clinical assessment and provided complete data on alcohol use and internalising disorders. Table 1 shows that 9.7% of the cohort presented with GAD,10.9% presented with depression and 1% with FBA. There were differences in GAD (11.6 vs 6.5%, p<0.001), depression (13.4% vs 6.9%, p<0.001) and FBA (1.3% vs 0.5%, p=0.02) by sex, with women indicating more internalising disorders than men. Overall, for alcohol use, 379 participants (10.6%) met criteria for harmful drinking with women more likely to be lower-risk drinkers compared with men (44.8% vs 32.2%) while men were more likely to be hazardous drinkers (53.7% vs 46.75) and harmful (14% vs 8.5%) drinkers (p<0.001).

Table 2 shows an association between alcohol consumption and internalising problems with harmful drinkers indicating more GAD (13.46% vs 11.39%), depression (17.68% vs12.58%) and FBA (2.37% vs 1.19%) compared with lower-risk drinkers. Harmful drinking, after adjustment for sex and socioeconomic status, was associated with a higher prevalence of depression (OR 1.8, 95% CI 1.3 to 2.4, p<0.001), anxiety (OR 1.4, 95% CI 1.0 to 2.0, p<0.001) and FBA (OR 2.4, 95% CI 1.04 to 5.56, p=0.009) compared with lower-risk drinkers. In contrast, hazardous drinkers presented with a lower prevalence of GAD (7.55%), depression (8.17%) and FBA (0.62%) compared with harmful and lower-risk drinkers. After adjustment, hazardous drinking was associated with a lower prevalence of GAD (OR 0.69, 95% CI 0.54 to0.88) and depression (OR 0.68, 95% CI 0.54 to 0.86) but not FBA (OR 0.59, 95% CI 0.27 to 1.27) compared with lower-risk drinkers. We found similar results when analysing alcohol consumption and individual, non-co-morbid internalising disorders, and these results are presented in online supplementary appendix 3.

Lastly, to explore the robustness of our findings with an alternative classification of alcohol misuse, a sensitivity analysis was conducted in which we found little difference to the distribution of alcohol use and misuse by sex and by internalising symptoms (online supplementary appendix 4).

## DISCUSSION

### Main findings

Our main findings show an association between all internalising disorders (GAD, depression and FBA) and harmful alcohol consumption with harmful drinkers more likely to present with GAD (14% vs 11%), depression (18% vs 13%) and FBA (2% vs 1%) compared with lower-risk drinkers. After adjustment for sex and socioeconomic status, harmful drinkers were more likely to present with anxiety (by 40%), depression (by 80%) and FBA (by 141%) compared with lower-risk drinkers. We found a J-shaped distribution with lower rates of internalising disorders particularly GAD (OR 0.69, 95 CI 0.54 to 0.88) and depression (OR0.68, 95% CI 0.54 to 0.86) in hazardous drinkers compared with lower-risk drinkers.

### Comparison with previous studies

Depression was more marked in women (13.4%) than in men(6.9%) consistent with previous work on sex differences in depression in early adulthood.^2 26 27^ Similarly, anxiety was more marked in women (11.6%) than in men (6.5%). Although anxiety and depression are comorbid conditions which share genetic markers, neural mechanisms and treatment effects,^28^ each of these internalising disorders have individual characteristics that correspond different to the use and misuse of alcohol as our results depict.

Previous studies have reported higher levels of internalising disorders both in non-drinkers and harmful drinkers using AUD from the DSM-IV criteria.^29–31^ Some studies suggest a dose–response relationship between internalising symptoms and alcohol consumption^6^ while others indicate a consistent, elevated relationship across all levels of alcohol use.^32^ In contrast, we found a potential J-shaped relationship between alcohol use and internalising symptoms in young adults with hazardous drinking associated with lower levels of internalising compared with lower-risk drinkers and harmful drinkers.

A Brisbane-based maternal cohort found similar J-shaped relationships between alcohol consumption and internalising disorders at age 30 years and linear relationships at ages 25 and 40 years,^33^ consistent with previous work particularly pertaining to Australian men.^34^ Results from a New Zealand-based 35-year birth cohort showed little evidence of J-shaped relationship over the life course.^7^ Inverse relationships of higher mental ill-health in lower-risk drinkers or abstainers compared to harmful drinkers defined using DSM-IV criteria have also been reported,^33 35^ adding greater complexity to understanding the nature of this relationship across varying levels of alcohol consumption. It has been suggested that the increased risk of depression and anxiety found among both abstainers and those who consume large amounts of alcohol (harmful drinkers) may be an indicator of poor overall health,^33^ or ‘sick-quitters’,^36^ that is, individuals who have experienced psychiatric disorders as a result of their alcohol consumption and quit drinking—which may explain a higher prevalence of depression and anxiety in the lower-risk drinking group.^37^ Other studies with longitudinal data found that abstinence may be a true predictor of poor mental health and is an early indicator of the relationship between abstinence and mortality.^38^ Other potential explanations for the association between hazardous drinking and lower rates of internalising disorders include an increasing social or peer interaction as part of the UK’s wider ‘drinking culture’,^3^ which may mitigate or preclude the effects of mental ill-health in a young adult population. Personality traits and temperament may also explain behaviour with alcohol as novelty seeking, harm avoidance and reward dependence on consuming alcohol may shape the way in which AUD develops.^39 40^ This may be unique to adolescents and young adults as these periods are often associated with higher prevalence of alcohol use and associated problems, but this transitional period is also characterised by normative personality changes leading to psychological maturity and increasing emotional stability.^41^ It could also be that lower-risk drinkers may have differing social characteristics (although we adjust for socioeconomic status and the association persists),^7^ or simply unmeasured confounding such as genetic predisposition to alcohol tolerance.^42^

### Limitations

The cross-sectional nature of the study design is a limitation as we cannot determine the nature or direction of associations between alcohol use and internalising disorders. Researchers^6 7^ also suggest that J-shaped relationships between these are largely reported from cross-sectional study designs while longitudinal analyses may better capture the variation of alcohol consumption and associated internalising disorders across the life course. Second, attrition of participants and missing data from several waves of data collection is an issue for any longitudinal cohort such as ALSPAC. When we compared clinic attenders with non-attenders (online supplementary appendix 2), we found differences in sex, social status, income levels and maternal education. More women attended clinic (62.6%) than did not in the non-attender group (44.83%) and in those who attended, 45.4% of achieved educational qualifications beyond school while this was only 27.9% of the non-attenders. In order to mitigate some of these differences, we used multiple imputation techniques using a large number of auxiliary variables in our analyses. While this does not completely address the high attrition rate in ALSPAC, it does help maximise the model power. Third, the CIS-R which was used to determine internalising symptoms focuses on the past 1 week only and may not include on all internalising disorder ‘cases’. However, CIS-R has demonstrated good validity and reliability in diagnosing common mental disorders and depressive episodes according to ICD-10 and compares well with other self-administered tools such as the General Health Questionnaire and Centre for Epidemiologic Studies Depression Scale.^43^ Lastly, the analysis excludes additional covariates factors such as personal relationships or exposures to early childhood adversities that may independently explain the presence of internalising disorders^44^ and alcohol misuse,^45^ but may also mediate the associations between these disorders.^46^

### Implications

The results suggest that young adults who drink harmfully do present with increased internalising symptoms, and future work should focus on unpicking the complex and reverse relationships found between hazardous drinking and harmful drinking and, internalising disorders. The J-shaped results suggest a varying relationship between different levels of alcohol consumption and internalising disorders being subject to unmeasured covariates (eg, personality temperaments or genetic predisposition or exposure to early childhood adversities) and relationship itself further varying across the life course. Future work should consist of longitudinal analyses where the nature and direction of the relationship is explored with specific consideration for personal- and societal-level factors.

## Supplementary Material

Supplementary Material1

Supplementary Material2

Supplementary Material3

Supplementary Material4

Supplementary Material5

## Figures and Tables

**Table 1:** The prevalence of generalised anxiety disorder (GAD), depression and fear-based anxieties and alcohol phenotypes by sex

24-year clinic (TF5) n=3572	Anxiety GAD (n, %)	Depression (n, %)	Fear-based anxieties* (n, %)	Alcohol phenotype** (n, %)		
				0	1	2
Overall	347 (9.7%)	391 (10.9%)	37 (1.0%)	1431 (40.1%)	1762 (49.3%)	379 (10.6%)
Female (n=2236)	260 (11.6%)	299 (13.4%)	30 (1.3%)	1001 (44.8%)	1044 (46.7%)	191 (8.5%)
Males (n=1336)	87 (6.5%)	92 (6.9%)	7 (0.5%)	430 (32.2%)	718 (53.7%)	188 (14.1%)
χ^2^, p value	25, p<0.001	36.1, p<0.001	5.5, p=0.02	65.6, p<0.001		

* Fear-based anxieties is an amalgamation of panic disorder, agoraphobia and social phobia.

** Alcohol phenotype groups: 0=lower-risk drinkers, 1=hazardous drinkers, 2=harmful drinkers.

**Table 2:** Logistic regression model outputs based on imputed data set when comparing hazardous drinkers and harmful drinkers to lower-risk drinkers

24-year clinic	Prevalence (n, %)	Crude (model 1) OR (95% CI, p value)	Model 2* OR (95% CI, p value)	Model 3* OR (95% CI, p value)
Anxiety, GAD				
Lower-risk drinkers	163 (11.39)	–	–	–
Hazardous drinkers	133 (7.55)	0.64 (0.50–0.80)	0.67 (0.53–0.86)	0.69 (0.54–0.88)
Harmful drinkers	51 (13.46)	1.20 (0.86–1.69) p<0.001	1.36 (0.97–1.91) p<0.001	1.39 (0.99–1.96) p<0.001
Depression				
Low-risk drinkers	180 (12.58)	–	–	–
Hazardous drinkers	144 (8.17)	0.62 (0.49–0.79)	0.66 (0.52–0.84)	0.68 (0.54–0.86)
Harmful drinkers	67 (17.68)	1.49 (1.09–2.02) p<0.001	1.72 (1.26–2.36) p<0.001	1.77 (1.29–2.43) p<0.001
Fear-based anxieties				
Low-risk drinkers	17 (1.19)	–	–	–
Hazardous drinkers	11 (0.62)	0.52 (0.24–1.12)	0.57 (0.27–1.22)	0.59 (0.27–1.27)
Harmful drinkers	9 (2.37)	2.02 (0.89–4.58) p=0.01	2.40 (1.05–5.47) p=0.006	2.41 (1.04–5.56) p=0.009

* Model 1 (crude). Model 2 adjusted for sex. Model 3 adjusted for sex, social status, income and maternal education.

** Estimates are accompanied by omnibus p values with two df to assess differences in odds across all alcohol groups.

## What is already known on this subject

- Depression and anxiety often associate with harmful alcohol consumption. Early adulthood is a developmental period marked by significant life changes and is considered a high-risk period for the onset of both, internalising disorders and alcohol-related problems. Internalising disorders and alcohol problems have a bidirectional relationship and have both been associated with longer-term mental ill-health and alcohol misuse.

## What this study adds

- The study reports that young adults, from a UK-based birth cohort (ALSPAC), who drink harmfully, are more likely to have internalising disorders such as depression, anxiety and fear-based anxieties. Women report more internalising disorders than men. After adjusting for sex and socioeconomic status, harmful drinking was associated with a higher prevalence of internalising disorders compared with lower-risk drinkers. However, future research should focus on unpicking the complex and reverse relationships found between hazardous drinking and harmful drinking and, internalising disorders.
