# Supplementary material for: Alcohol consumption and internalising disorders in young adults of ALSPAC: a population-based study
Source: J Epidemiol Community Health. Author manuscript; Available in PMC 2022 Mar 1. (PMC8886795; doi:10.1136/jech-2020-213922)
Supplement: Supplementary Material1 [file NIHMS1780577-supplement-Supplementary_Material1.pdf]

Supplementary Material 1

ALSPAC Sample Details

Pregnant women residing in the southwest of England with an estimated date of delivery between April 1, 1991, and December 31, 1992, were invited to participate. The initial number of pregnancies enrolled is 14,541 (for these at least one questionnaire has been returned or a “Children in Focus” clinic had been attended by 19/07/99). Of these initial pregnancies, there was a total of 14,676 foetuses, resulting in 14,062 live births and 13,988 children who were alive at 1 year of age. For full details of the cohort profile and study design, see Boyd et al., (2013); and Fraser et al., 2013).

Study data were collected and managed using REDCap electronic data capture tools hosted at the University of Bristol (Harris et al., 2009; Harris et al., 2019). REDCap (Research Electronic Data Capture) is a secure, web-based software platform designed to support data capture for research studies, providing 1) an intuitive interface for validated data capture; 2) audit trails for tracking data manipulation and export procedures; 3) automated export procedures for seamless data downloads to common statistical packages; and 4) procedures for data integration and interoperability with external sources.

Please note that the study website contains details of all the data that is available through a fully searchable data dictionary and variable search tool (http://www.bris.ac.uk/alspac/researchers/our-data)

References:

Boyd, A., Golding, J., Macleod, J., et al., Cohort Profile: the 'children of the 90s'--the index offspring of the Avon Longitudinal Study of Parents and Children. *International journal of epidemiology*, 2012; *42*(1), 111-27.

Fraser, A., Macdonald-Wallis, C., Tilling, K.. Cohort Profile: the Avon Longitudinal Study of Parents and Children: ALSPAC mothers cohort. *International journal of epidemiology*, 2013; *42*(1), 97–110. doi:10.1093/ije/dys066

Northstone K, Lewcock M, Groom A *et al.* The Avon Longitudinal Study of Parents and Children (ALSPAC): an update on the enrolled sample of index children in 2019 [version 1; peer review: 2 approved]. *Wellcome Open Res* 2019, **4**:51 (https://doi.org/10.12688/wellcomeopenres.15132.1)

Harris, P. A., Taylor, R., Thielke, R.Research electronic data capture (REDCap)--a metadata-driven methodology and workflow process for providing translational research informatics support. *J Biomed Inform, 2019; 42*(2), 377-381. doi:10.1016/j.jbi.2008.08.010

Harris, P. A., Taylor, R., Minor, B. L., et al., The REDCap consortium: Building an international community of software platform partners. *J Biomed Inform, 2019; 95*, 103208. doi:10.1016/j.jbi.2019.103208
