# Supplementary material for: Alcohol consumption and internalising disorders in young adults of ALSPAC: a population-based study
Source: J Epidemiol Community Health. Author manuscript; Available in PMC 2022 Mar 1. (PMC8886795; doi:10.1136/jech-2020-213922)
Supplement: Supplementary Material2 [file NIHMS1780577-supplement-Supplementary_Material2.pdf]

Appendix 2: Characteristics of clinic non-attenders at 24 years.

**Characteristics**	**Attenders (n=3572)**	**Non-Attenders (n=5897)**	**Chi^2 result (p value)**
Sex (F, %)	2236 (62.6%)	2644 (44.83%)	279.8, p<0.001
Social Status (highest (%) and lowest (%))	101 (2.8%), 611 (17.1%)	303 (5.1%), 527 (8.9%)	167.6, p<0.001
Income (highest (%) and lowest (%))	788 (22%), 387 (10.8%)	755 (12.8%), 885 (15%)	148.5, p<0.001
Maternal Education (n (%) with more than O level, n with less than O level (%))	1620 (45.35), 555 (15.5%)	1648 (27.9%), 1604 (27.2%)	310.5, p<0.001
