# Supplementary material for: Alcohol consumption and internalising disorders in young adults of ALSPAC: a population-based study
Source: J Epidemiol Community Health. Author manuscript; Available in PMC 2022 Mar 1. (PMC8886795; doi:10.1136/jech-2020-213922)
Supplement: Supplementary Material3 [file NIHMS1780577-supplement-Supplementary_Material3.pdf]

Supplementary Material 2

Multiple Imputation

We used a complete case analysis for our primary findings and used an imputed sample (n=3572) to determine missing socioeconomic status data at 24 years. We used multiple imputation by chained equations (MICE) to replace missing socioeconomic status data. We assumed missingness was dependent on observed data from previous time points and missing at random. We imputed 100 data sets. To predict missing data, we used all variables selected for the analysis models and several auxiliary socio-demographic and mental health variables predictive of incomplete variables and/or missingness such as parity and mum's use of cannabis, alcohol and cigarettes. Monte Carlo errors were less than 10% of the standard error and the t values were less than 0.1. We were able to impute up to a sample of 3572 participants for whom there were complete measures of internalising outcomes at 24 years.

Analyses were then run across imputed datasets using the 'mi estimate' command which fits a model to each of the imputed data set and pools individual results using Rubin's combination rules (Sterne *et al.,* 2009; White *et al.,* 2011).

Sterne, J. A. C., White, I. R., Carlinet al. Multiple imputation for missing data in epidemiological and clinical research: potential and pitfalls. *British Medical Journal, 2009; 338*, b2393. doi:10.1136/bmj.b2393

White, I. R., Royston, P., & Wood, A. M.. Multiple imputation using chained equations: Issues and guidance for practice. *Stat Med, 2011; 30*(4), 377-399. doi:10.1002/sim.4067
