# Supplementary material for: Alcohol consumption and internalising disorders in young adults of ALSPAC: a population-based study
Source: J Epidemiol Community Health. Author manuscript; Available in PMC 2022 Mar 1. (PMC8886795; doi:10.1136/jech-2020-213922)
Supplement: Supplementary Material4 [file NIHMS1780577-supplement-Supplementary_Material4.pdf]

Appendix 3. The overlap of internalising disorders in the 24-year ALSPAC cohort

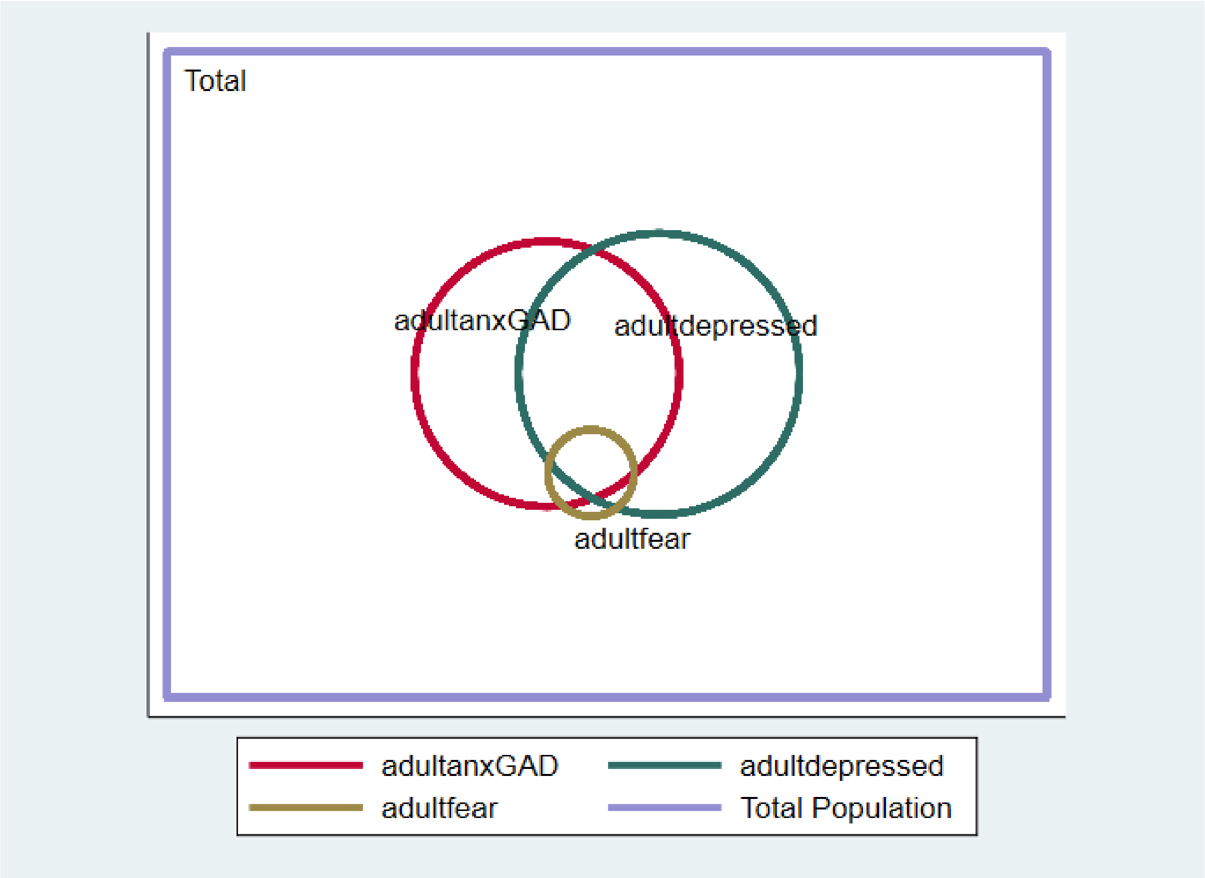

A: adultanxGAD: 347
B: adultdepressed: 391
C: adultfear: 37
AB Overlap: 183
AC Overlap: 31
BC Overlap: 29
ABC Overlap: 26
Total population: 357

Table showing the results of a multinomial logistic regression model when individual internalising disorders were considered as binary outcomes for hazardous drinkers and harmful drinkers compared with lower-risk drinkers.

**24-year clinic (TF5) n=3572**	Prevalence n (%)	Crude (model 1) OR (95% CI)	Model 2* OR (95% CI)	Model 3* OR (95% CI)
Lower-risk drinkers		1.00 ref	1.00 ref	1.00 ref
Hazardous drinkers				
No diagnosis	1545 (51.26)	1.00 ref	1.00 ref	1.00 ref
GAD only	71 (4.03)	0.83 (0.59-1.16)	0.86 (0.62-1.21)	0.88 (0.60-1.30)
Depression only	81 (4.60)	0.72 (0.53-0.97)	0.77 (0.56-1.05)	0.86 (0.60-1.21)
GAD and Depression only	54 (3.06)	0.55 (0.38-0.78)	0.58 (0.40-0.83)	0.55 (0.36-0.83)
GAD, Depression and FBA	7 (0.40)	0.47 (0.18-1.20)	0.52 (0.20-1.33)	0.46 (0.14-1.56)
Harmful drinkers				
No diagnosis	292 (9.69)	1.00 ref	1.00 ref	1.00 ref
GAD only	19 (5.01)	1.04 (0.61-1.75)	1.14 (0.67-1.91)	1.23 (0.69-2.2)
Depression only	34 (8.97)	1.47 (0.97-2.21)	1.69 (1.11-2.56)	1.61 (0.99-2.61)
GAD and Depression only	25 (6.60)	1.22 (0.77-1.95)	1.37 (0.85-2.19)	1.32 (0.76-2.30)
GAD, Depression and FBA	7 (1.85)	2.22 (0.86-5.69)	2.72 (1.05-7.03)	4.35 (1.54-12.27)

**Model 1 (crude). Model 2 adjusted for sex. Model 3 adjusted for sex, social status, income and maternal education*

No diagnosis (none of depression, GAD, FBA) (n=3014)
Gad only (n = 159)
Depression only (n = 205)
GAD and Depression only (n = 157)
GAD, Depression and FBA (n=26)
Some combinations not shown due to small cell-counts: FBA alone (n ≤ 5), GAD and FBA (n ≤ 5), depression and FBA (n ≤ 5).
**figures from multinomial logistic regression modelling
