# Supplementary material for: Alcohol consumption and internalising disorders in young adults of ALSPAC: a population-based study
Source: J Epidemiol Community Health. Author manuscript; Available in PMC 2022 Mar 1. (PMC8886795; doi:10.1136/jech-2020-213922)
Supplement: Supplementary Material5 [file NIHMS1780577-supplement-Supplementary_Material5.pdf]

Appendix 4 - SENSITIVITY ANALYSIS RESULTS

The prevalence of generalised anxiety disorder (GAD), depression and fear-based anxieties and alcohol phenotypes by sex using new alcohol thresholds

**24-year clinic (TF5) n=3572**	Anxiety GAD (n, %)	Depression (n, %)	Fear-based Anxieties~ (n, %)	Alcohol Phenotype (n, %) 0	1	2
Overall	347 (9.7%)	391 (10.9%)	37 (1.0%)	1431 (40.06%)	1710 (47.87%)	431 (12.07%)
Female (n=2236)	260 (11.6%)	299 (13.4%)	30 (1.3%)	1001 (44.77%)	1020 (45.62%)	215 (9.62 %)
Males (n=1336)	87 (6.5%)	92 (6.9%)	7 (0.5%)	430 (32.71%)	690 (51.65%)	216 (16.17%)
chi2, p value	25, *p*<0.001	36.1, *p*<0.001	5.5, *p*=0.02		69.15, *p*<0.001	

~ Fear-based anxieties is an amalgamation of panic disorder, agoraphobia and social phobia
**Alcohol phenotype groups: 0= lower-risk drinkers, 1=hazardous drinkers, 2=harmful drinkers

SENSITIVITY ANALYSIS RESULTS

Logistic regression model outputs based on imputed dataset when comparing hazardous drinkers and harmful drinkers to lower-risk drinkers using revised alcohol thresholds.

**24-year clinic**	Prevalence rates (n, %)	Crude (model 1) OR (95% CI, *p* value)	Model 2* OR (95% CI, *p* value)	Model 3* OR (95% CI, *p* value)
**Anxiety, GAD**				
Lower-risk drinkers	163 (11.39)	-	-	-
Hazardous drinkers	129 (7.55)	0.64 (0.50-0.80)	0.67 (0.53-0.86)	0.66 (0.50-0.88)
Harmful drinkers	55 (12.76)	1.14 (0.82-1.57)	1.28 (0.92-1.78)	1.36 (0.94-1.97)
		p<0.001	*p<0.001*	*p<0.001*
**Depression**				
Low risk drinkers	180 (12.58)	-	-	-
Hazardous drinkers	140 (8.19)	0.62 (0.49-0.79)	0.66 (0.52-0.84)	0.68 (0.54-0.86)
Harmful drinkers	71 (16.47)	1.37 (1.01-1.84)	1.59 (1.17-2.15)	1.63 (1.15-2.30)
		p<0.001	*p<0.001*	*p*<0.001
**Fear-based Anxieties**				
Low risk drinkers	17 (1.19)	-	-	-
Hazardous drinkers	11 (0.62)	0.49 (0.22-1.07)	0.53 (0.24-1.17)	0.49 (0.19-1.23)
Harmful drinkers	9 (2.37)	1.98 (0.89-4.35)	2.36 (1.05-5.23)	3.19 (1.36-7.44)
		p=0.009	*p*=0.001	*p*=0.001

**Model 1 (crude). Model 2 adjusted for sex. Model 3 adjusted for sex, social status, income and maternal education*

~Estimates are accompanied by omnibus p-values with two degrees of freedom to assess differences in odds across all alcohol groups
